# Alaska Native Elder-Centered Research Methodology

**DOI:** 10.1093/geroni/igab046.896

**Published:** 2021-12-17

**Authors:** Rosellen Rosich, Maria Crouch, Jordan Lewis

**Affiliations:** 1 University of Alaska Anchorage, Anchorage, Alaska, United States; 2 Yale School of Medicine, Department of Psychiatry , East Haven, Connecticut, United States; 3 Memory Keepers Medical Discovery Team/University of Minnesota Medical School, Duluth campus, Duluth, Minnesota, United States

## Abstract

Alaska Native (AN) Elders have historically been underrepresented in research. Innovative AN research posits that practice-based evidence is fundamental to culturally grounded, multifaceted methods. AN Elders is a cultural convention distinguishing Elders who continue to serve as an integral part of their family and community and recognized by their community as role models. Several studies will be discussed which employed Elders at every level of the research, ensuring cultural relevancy, outcomes, and dissemination activities. The findings lay the foundation for an Elder-centered research methodology that can be adapted and applied in other studies to encourage engagement of older adults. This methodology has potential to impact research for underrepresented groups and to rethink and reshape Western-centric practices. Findings from this research provides best practices for capacity building and sustainability, strategies for empowerment and prevention, and a framework for supporting the AN community in all phases of research.

